# A Delayed Anatomic Diagnosis and Management Challenge in an Initially Asymptomatic Infant With Type II Pulmonary Artery Sling: A Case Report

**DOI:** 10.3389/fcvm.2021.743848

**Published:** 2021-10-21

**Authors:** Xiaoqing Shi, Chuan Wang, Yimin Hua, Xiaoliang Liu, Hongyu Duan

**Affiliations:** ^1^Department of Pediatrics, West China Second University Hospital, Sichuan University, Chengdu, China; ^2^The Cardiac Development and Early Intervention Unit, West China Institute of Women and Children's Health, West China Second University Hospital, Sichuan University, Chengdu, China; ^3^Key Laboratory of Birth Defects and Related Diseases of Women and Children (Sichuan University), Ministry of Education, Chengdu, China; ^4^Key Laboratory of Development and Diseases of Women and Children of Sichuan, West China Second University Hospital, Sichuan University, Chengdu, China

**Keywords:** pulmonary artery sling, tracheal stenosis, bronchoscopy, delayed diagnosis, management

## Abstract

Pulmonary artery sling (PAS) is a rare but fatal malformation. Patients with PAS tend to develop obstructive symptoms in few weeks of life. Conversely, some patients may be otherwise mild or asymptomatic in their early life. Currently, no consensus on the intervention timing and treatment strategy for asymptomatic and mild cases has been reached. Moreover, the extent of tracheal stenosis is another determining factor for the choice of intervention timing since clinical symptoms might not correspond well with the degree of stenosis. Lack of comprehensive assessment of entire airways confer underestimation of disease severity and in turn improper choice of treatment regimens and poor outcomes. Herein, we described an infantile case of PAS, who was scheduled initially for periodic outpatient follow-up on account of the absence of symptoms and inadequate imaging assessment at diagnosis. The patient developed recurrent wheezing and progressive respiratory distress at 7 months of age. After left pulmonary artery (LPA) reimplantation without tracheal intervention, bronchoscopy was performed due to failure to wean from mechanical ventilation, which demonstrated complete tracheal cartilage rings, a long segment tracheal stenosis, a low tracheal bifurcation at T6, and the absence of a separate right middle lobe bronchus. The patient was finally diagnosed with type IIb PAS and extubated successfully following conservative treatment. Miserably, neurological sequelae were devastating, leading to poor outcomes. Comprehensive airway evaluation using bronchoscopy is substantial to early identification of all components responsible for airway compromise in PAS anatomic subtypes. Considering severe concomitant maldevelopment of the bronchial tree in children with type IIb PAS, early and complete correction by surgery might decrease perioperative morbidities and mortalities of these patients.

## Introduction

Pulmonary artery sling (PAS) is a rare but fatal malformation where the aberrant left pulmonary artery (LPA) arises from the right pulmonary artery (RPA), acting as a partial vascular ring (VR) around the trachea (1). Patients with PAS tend to develop obstructive symptoms in few weeks of life (2). Conversely, some patients may be mild or asymptomatic in their early life (3). Symptomatic PAS is recommended to receive early surgical intervention. However, no consensus on the intervention timing and treatment strategy for asymptomatic and mild cases has been reached. Regular follow-up for asymptomatic and mild cases is advocated by most centers due to the relatively encouraging results with conservative management and the evidence of increased risk regarding postoperative death or complications (4, 5).

Except for the presence or absence of clinical symptoms, the extent of tracheal stenosis is another determining factor for the choice of intervention timing since clinical symptoms might not correspond well with the degree of stenosis (6, 7). Notably, most clinicians, particularly those who are not cardiologists or respiratory specialists, would be not aware that the tracheal stenosis is not only related to the extrinsic local impression of the aberrant pulmonary artery on the trachea but also concomitant maldevelopment of the bronchial tree, which is often observed in type II of PAS. Therefore, it is common in the clinic that CT and MRI are considered to be sufficient for evaluating the presence of tracheal stenosis in patients with PAS. Consequently, the direct luminal assessment of entire airways by bronchoscopy is always ignored, especially in asymptomatic and mild cases, causing underestimation of disease severity and in turn improper choice of treatment regimens and poor outcomes.

Herein, we presented an infantile case of PAS, who was scheduled initially for periodic outpatient follow-up on account of the absence of symptoms and inadequate imaging assessment at diagnosis. The patient developed recurrent wheezing and progressive respiratory distress at 7 months of age. After LPA reimplantation without tracheal intervention, bronchoscopy was performed due to failure to wean from mechanical ventilation, which demonstrated complete tracheal cartilage rings, a long segment tracheal stenosis, a low-lying carina, and the absence of a separate right middle lobe bronchus. The patient was finally diagnosed with type IIb PAS and extubated successfully following conservative treatment. Miserably, the neurological sequelae were devastating, leading to poor outcomes. We aimed to share some experience and offer some references for the management strategies of initially mild asymptomatic cases with PAS.

## Case Description

A 9-month-old female infant with a bodyweight of 8.3 kg was admitted to our cardiac intensive care unit (CICU) due to failure to wean from invasive mechanical ventilation for respiratory failure. The patient was the second child of a young couple, without a history of consanguinity or a family history of vascular abnormalities. The fetal ultrasound at 35 weeks and 2 days of gestation revealed LPA arising from the RPA resulting in a PAS. Prenatal laboratory investigations were unremarkable including a normal amniotic fluid genetic microarray. Due to the prenatal diagnosis of PAS, the baby was delivered by cesarean section at 39 weeks of gestation with a birth weight of 3,000 g and normal Apgar scores.

**Figure 1:**
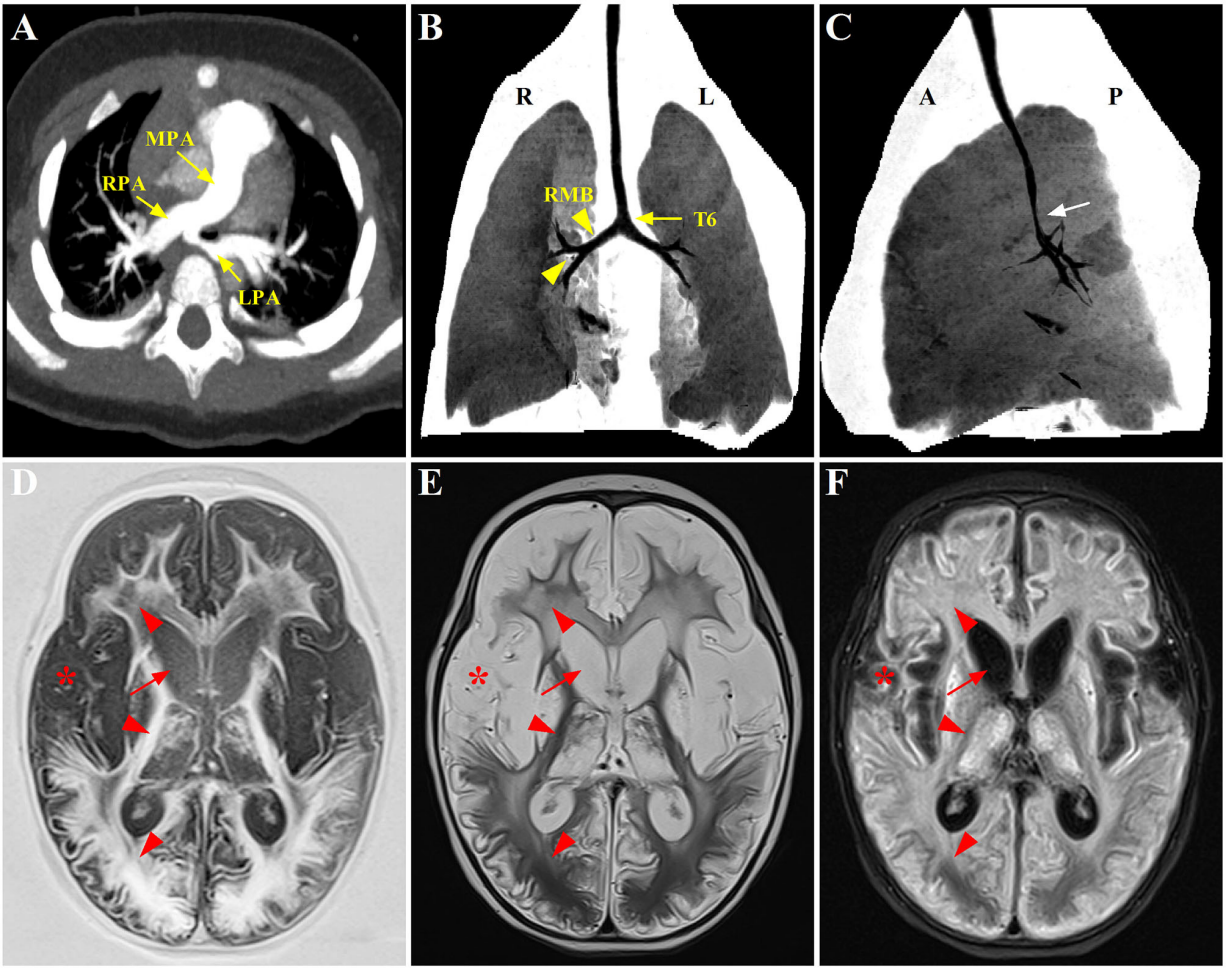
Radiographic evaluation of the PAS, tracheobronchial abnormalities, and neurological lesions through CT and MRI scans. **(A)** Preoperative CTA showing the anomalous origin of LPA from the posterior aspect of RPA. **(B,C)** Postoperative three-dimensional reconstruction of CT showing a lower level of bronchial bifurcation at T6 (yellow arrow) and a long RMB with bronchial divisions identical to left bronchial type (yellow arrowhead), associated with a stenotic segment in the distal trachea (white arrow); **(D–F)** Axial T1-weighted, T2-weighted, and T2-FLAIR brain MRI, respectively, displaying symmetrical signal abnormalities throughout the gray matter, white matter, and deep nuclei (red arrowhead), and extensive cortical necrosis (red asterisk), cerebrum atrophy and ventriculomegaly (red arrow). PAS, pulmonary artery sling; CTA, computed tomography angiography; LPA, left pulmonary artery; RPA, right pulmonary artery; RMB, right main bronchus; FLAIR, fluid attenuated inversion recovery.

A neonatal transthoracic echocardiogram further confirmed the diagnosis of PAS without additional intracardiac anomalies. Since the baby was initially asymptomatic with normal clinical examination, no imaging modalities were performed, merely periodic outpatient follow-up being arranged in the local hospital. The patient was referred to our hospital for developing recurrent wheezing and progressive respiratory distress following acute respiratory infections since 7 months of age. There was no vomiting, feeding issues, and recurrent respiratory infections. To better delineate the anatomy of the PAS and to evaluate the airway, a computed tomography angiography (CTA) was undergone at 8 months of age, demonstrating that the LPA had its origin from RPA and coursed between the esophagus and trachea to the left lung hilum (shown in Figure 1A). Despite segmental stenosis of proximal LPA was observed with the smallest inner diameter of 1.7 mm, no marked tracheal stenosis and complete tracheal cartilage rings were identified. Subsequently, the patient was hospitalized. Physical examination revealed moderate distress (nasal flaring and subcostal retractions) and tachypnea with respiratory rates of 56 breaths/min in the absence of dysmorphic features, heart murmurs, or extremity edema. Given the poor response to medical therapy, the patient was finally referred for surgical correction of the PAS by reimplantation of the reconstructed LPA to the left side of the main pulmonary artery (MPA) under cardiopulmonary bypass, but without tracheoplasty.

Despite being hemodynamically stable under mechanical ventilation, the postoperative course of the patient was complicated with airway obstruction and hypercapnia. Repeat chest CT (three-dimensional reconstruction) on postoperative day 15 showed two bi-lobed lungs and anomalous lateralizing features of the right main bronchus (RMB), characterized by a more horizontal course and a long main bronchus. Additionally, it was noted that the trachea bifurcation occurred more distally at the level of T6 rather than T4-5, with the lumen diameter of 2.0 mm at the most stenotic part of the trachea. Moreover, several consolidative opacities in the inferior lobes of both lungs were found (shown in Figures 1B,C). However, no features of cardiac heterotaxy and spleen anomalies were found in the subsequent ultrasound scan. The patient experienced three unsuccessful extubation within 18 days in one of which cardiopulmonary resuscitation was performed because of cardiac arrest. Thereafter, the patient was transferred to the CICU in our hospital under invasive respiratory support.

On CICU admission, the patient was sedated with sulfentanil (0.04 μg kg^−1^ h^−1^) and midazolam (8 μg kg^−1^ min^−1^) continuous infusion. SIMV mode of mechanical ventilation was employed with a peak airway pressure (P_peak_) of 22 cm H_2_O, a positive end-expiratory pressure (PEEP) of 7 cm H_2_O, a respiratory rate of 25 breaths, and an inspired oxygen fraction of 40%. The patient was hemodynamically stable without inotropic support. Her body temperature was 38.4°C. Clinical assessment reflected normal first heart sound, normal thoracic expansion, no hypotension (BP: 87/55 mmHg), and no hypoxia (SaO_2_: 95% on ventilation). Except for rales on pulmonary auscultation, the rest of her physical examination was unremarkable. Initial laboratory investigations revealed increased leukocytes (18,100/μm) with 74.5% neutrophils and 5.4% lymphocytes, normal hemoglobin (11.7 g/dl), normal platelets (17.1 × 10^4^/μl), and elevated C-reactive protein (1.88 mg/dl). Liver/renal function, procalcitonin, myocardial troponin I, and electrolytes were normal. Results of respiratory culture (tracheal aspirates), chlamydia/mycoplasma antibody, PPD test, T-spot assay, serum glucan/galactomannan tests, and blood culture were negative. Arterial blood gas reported hypercapnia (pH 7.31, PaO_2_ 96.2 mmHg, PaCO_2_ 72 mmHg, and HCO_3_ 22.3 mmol/L). Serial ultrasounds of the brain and heart were unremarkable, except for a pressure gradient of 16 mmHg across the anastomosis between the LPA and MPA. The chest X-ray revealed multifocal overinflation and increased opacities in the right infrahilar region, in part reflecting obstructive emphysema and atelectasis.

**Figure 2:**
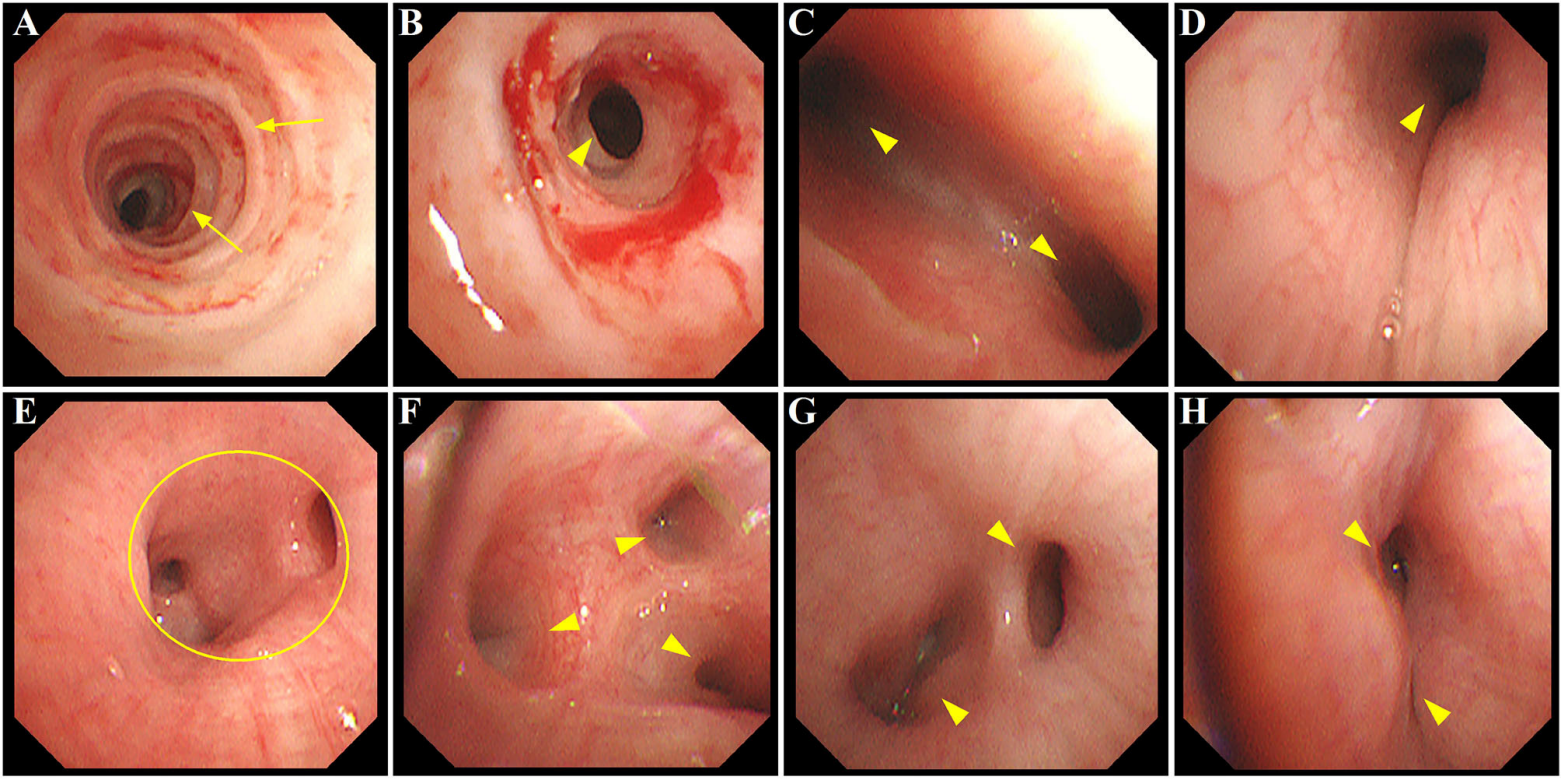
Bronchoscopy image showing extensive airway defects accompanied by mucosal hyperemia and edema. **(A,B)** long-segment stenosis (yellow arrowhead) with complete tracheal cartilage rings (O-rings) (yellow arrow) at the upper and lower trachea, respectively; **(C)** narrowing of the carina with increased angle between LMB and RMB (yellow arrowhead); **(D)** stenosis at RMB (yellow arrowhead); **(E)** absence of the orifice of RMLB at a level where the orifice of RLLB (yellow circle) normally existed; **(F)** stenosis at segmental bronchi beyond RMB (yellow arrowhead); **(G,H)** variable narrowing in subsegmental bronchi of LLLB (yellow arrowhead). LMB, left main bronchus; RMB, right main bronchus; RMLB, right middle lobe bronchus; RLLB, right lower lobe bronchus; LLLB, left lower lobe bronchus.

Imipenem (120 mg kg^−1^ d^−1^ divided every 6 h) and immunoglobulin (500 mg kg^−1^, single intravenous infusion) were given. To open the lung and prevent lung unit collapse, the patient was mechanically ventilated on SIMV mode, receiving a PEEP of 5–8 cm H_2_O with a tidal volume of 6–8 ml/kg. Three days later, the fever resolved, with an improvement of respiratory status. The patient was prepared for extubation with less sedation and a switch of ventilation mode from SIMV to PSV. However, a progressively increasing work of breathing was noted with noisy breathing and dramatical prolongation of the expiratory phase, a sign of airway obstruction and malacia. Ventilator-patient dyssynchrony was uncontrolled despite the adjustment of ventilatory parameters. A series of targeted tests were conducted to explore the underlying etiologies of ventilator dependence. First, systemic infections were not considered due to the absence of fever and negative results of blood routine, C-reactive protein, and procalcitonin. Second, new onset of lung infection, pulmonary edema, and plural effusion were excluded by a repeat chest radiography. Finally, unremarkable findings of ultrasounds did not support the diagnosis of pericardial effusion, low cardiac output syndrome, residual cardiac anomalies, intracranial hemorrhage/hypertension, and diaphragmatic weakness. Ultimately, a decision was made to assess the tracheal anatomy under general anesthesia. After preoperative preparation and anesthesia, electronic fiberoptic bronchoscopy was performed at the bedside of the patient on postoperative day 22. Complete tracheal cartilage rings were found from 1.5 cm below the glottis to 1.0 cm above the carina, with the stenotic length of 5.5 and 1.0 cm at the upper and lower stenotic segment, respectively (O-rings with the smallest inner diameter of 3.6 mm). Tracheal bifurcation occurred distally at the level of T6 with an inverted T-shape. No stenosis was seen in the left main bronchus (LMB); however, a long, stenotic, and collapsed RMB with only two distal bronchus orifices were observed (absence of a separate right middle lobe bronchus). Notably, multiple additional areas of variable narrowing in the subsegmental bronchi in both lungs were found. Additionally, extensive mucosal swelling with surrounding hyperemia and sticky secretions was prominent as well (shown in Figure 2). The assessment of tracheobronchomalacia was not available through the bronchoscopy, since spontaneous respiration was blocked by general anesthesia. Following bronchial washings, tests of the lavage fluid were performed, showing negative results for bacteria, fungi, viruses, protozoa, and malignant cells.

**Figure 3:**
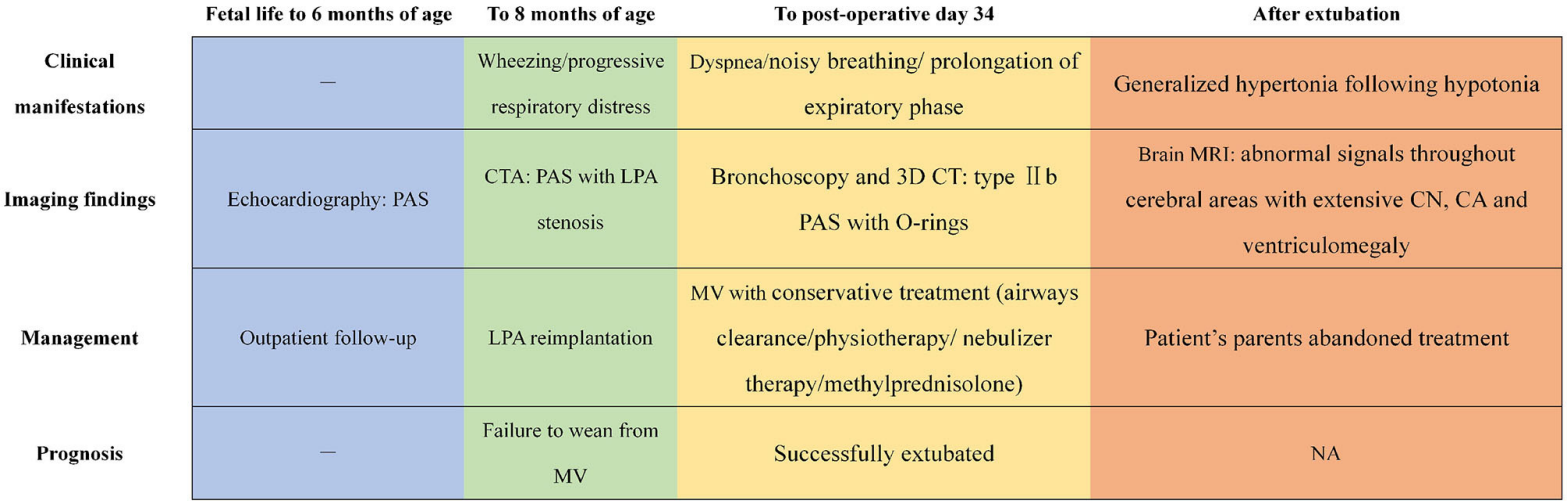
The timeline showcasing relevant data from the episode of care. PAS, pulmonary artery sling; CTA, computed tomography angiography; LPA, left pulmonary artery; MV, mechanical ventilation; 3D, three-dimensional; CN, cortical necrosis; CA, cerebrum atrophy; NA, not available.

These bronchoscopy findings were consistent with a diagnosis of type IIb PAS. Tracheoplasty was not performed, due to the economic factor and the possible related complications. Under conscious sedation, she was managed conservatively by effective clearance of airways, massive physiotherapy, and nebulizer therapy (albuterol, budesonide, and epinephrine). In addition, methylprednisolone (3 mg kg^−1^ d^−1^) was given intravenously for 3 consecutive days prior to extubation. The sedatives were gradually tapered and discontinued. Fortunately, the patient was successfully extubated on postoperative day 34. While the respiratory condition of this case continued to improve following non-invasive respiratory support, low muscle tone and strength in the extremities were identified later, indicating neurological dysfunction probably related to hypoxic injury postoperatively. Trajectories of hypotonia over several weeks followed by generalized hypertonia were highly predictive of persistent neurological sequelae. MRI was performed to evaluate brain lesions. Symmetrical abnormal signals were widely detected throughout the gray matter, white matter structures, and deep nuclei in association with extensive cortical necrosis, cerebrum atrophy, and ventriculomegaly (shown in Figures 1D–F). Finally, the parents of the patient abandoned treatment due to economic factors and took the baby home. Figure 3 summarized the timeline with relevant data from the episode of care in this case.

## Discussion

Pulmonary artery sling is one of the most lethal VRs presenting in neonates and infants with symptoms of respiratory compromise. Apart from the tightness of the ring, associated tracheobronchial anomalies also significantly determine morbidity and mortality (8, 9). Early and adequate recognition of these combined anomalies is clinically substantial to proper management and satisfactory long-term outcomes. In this report, we described a delayed diagnosis of the anatomic subtype and the resulting challenges of therapy in an infant with type IIb PAS. Poor diagnostic ability and lack of understanding of the disease were believed to have contributed to these results. This case highlighted not only the importance of focusing on comprehensive airway evaluation for the PAS subtype but also the management strategies for initially mild asymptomatic cases.

Since this lesion is mechanical in nature, surgical correction is, thus, the mainstay of treatment. The timing of such surgery highly depends on the severity of symptoms. Symptomatic patients with PAS typically require surgery to prevent serious complications resulting from hypoxic or apneic spells (6). However, controversy exists in when to offer surgical repair for asymptomatic or mildly symptomatic patients. Currently, no formal studies have addressed this question specifically. While it seems reasonable to propose clinical follow-up or medical treatment for their symptoms due to concerns about risks of cardiopulmonary bypass and postoperative complications (10), the indications are lacking, regarding the methods to evaluate those patients managed conservatively and the optimal timing to be dealt with surgically (2). Coupled with a progressive understanding of PAS, some authors have proposed early surgical correction under the following considerations. First, symptom onset of these cases is often triggered by acute infections or physical activity and maybe catastrophic, necessitating dependence on ventilator support and extracorporeal membrane oxygenation (2, 11, 12). In this setting, some cases are redirected to surgery in critical clinical status, with postoperative mortality being reported to be 50% as compared to 6.4% in its absence (9). Additionally, even without critical clinical conditions, symptomatic cases still have higher perioperative morbidities and mortalities in comparison to asymptomatic ones (13). Persistent airway compression might contribute to long-term tracheobronchomalacia and defective ventilation, resulting in persistence of airway symptoms and lung parenchymal hypoplasia despite relief of the compression (13, 14). Furthermore, it has been suggested that unrepaired VRs might be associated with neurocognitive problems (15). These findings provide some evidence that early surgery might be appropriate for asymptomatic or mildly symptomatic cases, especially for those complicated by tracheal deformation based on bronchoscopic and radiologic diagnosis (11, 16, 17). As for our case, the patient had PAS combined with severe tracheobronchial malformations in consistent with type IIb PAS. Low awareness of this subtype rendered delayed identification of its airway features. Surgical repair was not considered until after marked symptoms of airway compromise had been present. Considering that complete tracheal cartilage rings and long-segment tracheal stenosis were thought to be high-risk factors for poor prognosis (16, 18), the earlier operation might decrease the perioperative morbidity in this patient.

The options of surgical procedures for PAS are mainly based on the severity of combined tracheobronchial malformations. Although LPA reimplantation alone is preferable for type I cases, the treatment strategy for type II cases is still under debate due to the lack of sufficient evidence (7). Most authors suggest that LPA reimplantation plus simultaneous tracheoplasty should be performed in patients presenting moderate or more severe clinical symptoms (19–21). Conversely, there is a growing number of reports showing that LPA reimplantation without tracheoplasty confers good outcomes even in severe cases (4, 5, 22). Indeed, it is difficult to predict, before surgery, if the respiratory symptoms in patients with PAS and congenital tracheal stenosis (CTS) have to be ascribed to one, the other or, to the combination of both. Hence, in addition to clinical findings, the internal diameter and length of CTS were reported as more reliable indicators for surgical intervention, wherein a tracheal diameter <3.7 mm and/or a diameter/length ratio <5.9 were supposed to be an indication for tracheoplasty (4, 7, 8). In our case, on account of severe stenosis (3.6 mm at the narrowest diameter) extending to all the trachea seen on bronchoscopy, it was impossible to assume that the repair of PAS alone would be able to resolve her condition. This probably predisposed the patient to recurrent airway obstruction and long-term dependence on ventilator assistance, thereby causing severe neurological sequelae in the postoperative period. On the other hand, a dramatic evolution in recent surgical techniques has contributed to the excellent outcomes. Concomitant procedures to simultaneously correct vascular compression and all the airway defects by using slide tracheoplasty can prevent multiple reoperations with their attendant increased risks (9, 13), which would probably pose benefits to our case.

Bronchoscopy is essential in revealing the severity and extent of tracheal stenosis. It is superior in assessment of endoluminal airways, dynamic investigation of combined tracheal collapse (especially about distal malacia), and visualization of external compression on the trachea, and in identifying anatomical patterns of tracheal stenosis (18, 19). It is strongly recommended that all symptomatic patients requiring surgery should undergo bronchoscopy to define the features of airway abnormality or other unexpected pathology (6). However, its indication and timing for mild or asymptomatic patients are still controversial. Our patient was free of symptoms in the early months after birth but confirmed with severe tracheal stenosis using the bronchoscopic procedure postoperatively, suggesting clinical symptoms might not correspond well with the degree of stenosis. Additionally, although this case was proven to have congenital tracheal rings, such radiological features were not noted by an initial CT evaluation, indicating CT scan alone might underestimate the severity of the airway narrowing and not be sufficient to rule out CTS, especially for PAS with associated complete tracheal rings as already reported in the literature (20, 23). Importantly, except for a “fixed” airway narrowing, concomitant tracheobronchomalacia, which is characterized by an excessive “dynamic” airway collapse (more than 50% of airway lumen) on expiration diagnosed at bronchoscopy (24), has the ability to significantly and adversely affect survival (19). In these patients, preoperative tracheobronchomalacia (either a primary defect within the cartilages or secondary to extrinsic compression), if present and not addressed adequately, is frequently the main reason for postoperative morbidities and/or the need for re-operative procedures (19). Considering bronchoscopy is the most critical component of assessing children with suspicion of dynamic airway collapse, a missed diagnosis of tracheobronchomalacia in our case could be another reason for prolonged respiratory symptoms and failure to wean from medical ventilation. Discouragingly, despite postoperative identification of the PAS anatomic subtype, tracheobronchomalacia, and/or cartilage defect had never been addressed due to blockage of spontaneous respiration by general anesthesia and ignorance about the importance of bronchoscopic biopsy. On the basis of experience from the present case, early preoperative evaluation of the PAS anatomic subtype and its coexisting airway collapse would be beneficial even for initially mild or asymptomatic cases in terms of subsequent intervention decision and long-term outcomes. Apart from providing crucial information preoperatively, intraoperative bronchoscopy can recognize the site of tracheal stenosis, the extent of tracheal opening, and the adequacy of surgical repair (8, 19). Careful respiratory assessment is also essential to postoperative care, particularly for infants who have had surgery for severe symptoms since airway obstruction may persist for weeks or even months (6). In this context, a bronchoscopy is also a useful tool for the elective or urgent post-repair evaluation of granulation tissue, re-stenosis and scarring of the airways, and stability of the reconstructed trachea by following up the airway malacia evolution (19, 25).

At the moment, the precision and non-invasive nature render CT to be the best adjunct to bronchoscopy in terms of airway obstruction evaluation. Contrast-enhanced three-dimensional (3D) CT is particularly helpful for simultaneous evaluation of the airway anatomy and the neighboring vascular structures, which can be applied to newborns and infants and is pivotal for planning surgery (19). Personalized preoperative 3D-CT bronchography and angiography (3D-CTBA) helps to visualize the pulmonary vessel branches and bronchial structures, contributing to the safe and efficient performance of thoracic surgery (26, 27). The combination of 3D-CT bronchography with bronchoscopy has the advantage of demonstrating the airway distal to the stenosis, where it may be unsafe to advance the bronchoscope. Noticeably, without the use of sedation or anesthesia, paired images from a dynamic 4D-CT during physiological breathing are more sensitive to address bronchomalacia compared with bronchoscopy, avoiding underestimation of the disease extent or misdiagnosis of associated bronchomalacia as fixed stenosis (23, 28). As for our case, although 3D-CT provided images of the abnormal carina and right lobar bronchus, limited recognition of type IIb PAS hindered us from obtaining detailed information regarding bronchial sites of stenosis and relationships between the airways and neighboring vascular structures. Moreover, the diagnosis of tracheobronchomalacia was difficult due to the lack of preoperative bronchoscopic assessment along with the unavailability of dynamic 4D-CT. With the advances in both CT technology and pediatrician experience in developing countries, more patients with PAS will gain an early diagnosis and proper management in the future.

## Conclusions

Pulmonary artery sling is a rare but fatal abnormality. The tracheal stenosis is not only related to the extrinsic local impression of the aberrant pulmonary artery on the trachea but also concomitant maldevelopment of the bronchial tree. This consequently results in a poor prognosis. Comprehensive airway evaluation using bronchoscopy is substantial to early identification of PAS anatomic subtypes and all components responsible for airway compromise, especially for initially asymptomatic cases. Considering severe concomitant maldevelopment of the bronchial tree in children with type IIb PAS, early and complete correction by surgery might decrease perioperative morbidities and mortalities of these patients.

## Data Availability Statement

The original contributions presented in the study are included in the article/supplementary material, further inquiries can be directed to the corresponding author/s.

## Ethics Statement

Written informed consent was obtained from the patient's parents, for the publication of any potentially identifiable images or data included in this article.

## Author Contributions

HD contributed to conceptualization, writing, and editing. YH and XL performed data curation, methodology, and formal analysis. CW and XS were involved in supervision, validation, and writing assistance. All authors read and approved the manuscript.

## Funding

This study was supported by the National Natural Science Foundation of China (Grant Nos. 81971457 and 81800288), Science Technology Plan Projects of Applied Basic Research in Sichuan province (2020YJ0234), and Research Projects of Health and Family Planning Commission of Sichuan Province (No. 17PJ258).

## Conflict of Interest

The authors declare that the research was conducted in the absence of any commercial or financial relationships that could be construed as a potential conflict of interest.

## Publisher's Note

All claims expressed in this article are solely those of the authors and do not necessarily represent those of their affiliated organizations, or those of the publisher, the editors and the reviewers. Any product that may be evaluated in this article, or claim that may be made by its manufacturer, is not guaranteed or endorsed by the publisher.

## Abbreviations

PAS: pulmonary artery sling
LPA: left pulmonary artery
RPA: right pulmonary artery
VR: vascular ring
CICU: cardiac intensive care unit
CTA: computed tomography angiography
MPA: main pulmonary artery
RMB: right main bronchus
Ppeak: peak airway pressure
PEEP: positive end-expiratory pressure
CRP, LMB: left main bronchus
CTS: congenital tracheal stenosis
3D: three-dimensional
3D-CTBA: 3D-CT bronchography and angiography.
